# A phase II study of gemcitabine and docetaxel combination in relapsed metastatic or unresectable locally advanced synovial sarcoma

**DOI:** 10.1186/s12885-023-11099-4

**Published:** 2023-07-08

**Authors:** Ghazal Tansir, Sameer Rastogi, Akash Kumar, Adarsh Barwad, Asit R. Mridha, Ekta Dhamija, Shamim A. Shamim, Sushma Bhatnagar, Sandeep Bhoriwal

**Affiliations:** 1 Department of Medical Oncology, Dr. BRAIRCH, All India Institute of Medical Sciences, Ansari Nagar, New Delhi 110029 India; 2 Department of Medical Oncology, National Cancer Institute, All India Institute of Medical Sciences, New Delhi, 110029 India; 3 Department of Pathology, All India Institute of Medical Sciences, Ansari Nagar, New Delhi 110029 India; 4 Department of Radiodiagnosis, All India Institute of Medical Sciences, Ansari Nagar, New Delhi India 110029; 5 Department of Nuclear Medicine, All India Institute of Medical Sciences, Ansari Nagar, New Delhi India 110029; 6 Department of Oncoanesthesia and Palliative Medicine, Dr. BRAIRCH, All India Institute of Medical Sciences, Ansari Nagar, New Delhi India 110029; 7 Department of Surgical Oncology, Dr. BRAIRCH, All India Institute of Medical Sciences, Ansari Nagar, New Delhi 110029 India

**Keywords:** Soft tissue sarcoma, Palliative chemotherapy, Synovial sarcoma, Quality of life

## Abstract

Synovial sarcoma (SS) is one of the commonest non-rhabdomyosarcoma soft tissue sarcoma with limited treatment options in the relapsed and advanced settings. The combination of gemcitabine and docetaxel has demonstrated its role predominantly in leiomyosarcoma and pleomorphic sarcomas but has not been prospectively studied in SS. This trial assesses the efficacy, tolerability and quality of life (QoL) with this regimen in metastatic/unresectable locally advanced relapsed SS.

**Patients and methods** This was a single-arm, two-stage, phase II, investigator-initiated interventional study among patients with metastatic or unresectable locally advanced SS who had progressed after at least one line of chemotherapy. Gemcitabine 900 mg/m2 on days 1 and 8 and docetaxel 75 mg/m2 on day 8 were administered intravenously every 21 days. The primary endpoint was 3-month progression-free rate (PFR); overall survival (OS), progression-free survival (PFS), overall response rate (ORR), safety and quality of life (QoL) constituted the secondary endpoints.

**Results** Twenty-two patients were enrolled between March 2020 and September 2021 and the study had to be closed early due to slow accrual. The study population comprised of 18 (81.8%) patients with metastatic disease and 4 (18.2%) patients with locally advanced, unresectable disease. The most common primary sites of disease were extremity in 15 (68%) and the median number of lines of prior therapies received was 1 (range 1–4). 3-month PFR was 45.4% (95% CI 24.8–66.1) and ORR was 4.5%. Median progression-free survival (PFS) was 3 months (95% CI 2.3–3.6) and median OS was 14 months (95% CI 8.9–19.0). 7 (31.8%) patients experienced grade 3 or worse toxicities, including anemia (18%), neutropenia (9%) and mucositis (9%). QoL analysis demonstrated significant decline in certain functional and symptom scales, while financial and global health scales remained stable.

**Conclusion** This is the first prospective study on the combination of gemcitabine and docetaxel performed specifically in patients with advanced, relapsed SS. Although the accrual of patients could not be completed as planned, the therapy did produce clinically meaningful outcomes and met its primary endpoint of 3-month PFR. This result, along with the manageable toxicity profile and stable global health status on QoL analysis, should encourage further studies.

**Trial registration** This trial was prospectively registered under the Clinical Trials Registry of India on 26/02/2020 (Registration number: CTRI/2020/02/023612).

**Supplementary Information:**

The online version contains supplementary material available at 10.1186/s12885-023-11099-4.

## Background

Synovial sarcoma (SS) constitutes 5–10% of all soft tissue sarcomas (STS) [1] and is the most common non-rhabdomyosarcomatous STS in adolescents and young adults [2]. It is one of the predominant subtypes of STS in Indian patients, comprising 22.5% of all cases [3]. SS carries a high rate of local and metastatic recurrences [4], with the median overall survival (OS) and time to next treatment (TNT) being 19.7 months and 8.7 months respectively [5]. Though regarded as a chemosensitive tumor, the prognosis of metastatic disease remains limited, with 5-year OS approximately 10% in metastatic disease. The 5-year post-recurrence survival in SS varies from 67% in local recurrence to 0% in patients with multiple metastases [6].

Anthracyclines constitute the frontline treatment for advanced SS, with response rates of 16–27% demonstrated across all STS subtypes [7]. Beyond the first line, sequencing the other treatment options depends on individual patient-based considerations. These later-line treatments include pazopanib, high-dose ifosfamide and trabectedin. The current era of personalized medicine has led to the exploration of unique cancer testis antigens (CTAs) such as NY-ESO-1, MAGE-A4 and PRAME that can serve as therapeutic targets [8]. However, this use of adoptive immunotherapy in SS remains currently limited to patients having the HLA A*02:01 haplotype [9].

Gemcitabine and docetaxel (GD) form a synergistic cytotoxic combination when docetaxel is sequentially administered after gemcitabine, as demonstrated by Leu et al. in in-vitro and in-vivo analyses [10]. The efficacy of the Fixed Dose Rate (FDR) administration of gemcitabine (10 mg/m^2^/min) compared to the 30-min infusion has been found to be superior in both preclinical and clinical trials due to longer exposure to its active cytotoxic metabolite [11, 12]. Previous trials exploring the efficacy of GD among patients with soft tissue sarcomas have included less than 10% patients with SS. Hence, there is a lacuna in existing literature on the role of this combination in this particular disease. We conducted this study due to the high prevalence of SS especially in the Indian population and the paucity of treatment options in later-line setting.

## Methods

### Study design and participants

The study was designed as a two-stage, single arm phase II trial among patients with metastatic or locally advanced unresectable relapsed SS enrolled between March 2020 and September 2021. The eligible patients included those with histopathologically proven synovial sarcoma who had received at least one line of medical therapy and had progressed with unresectable locally advanced or metastatic disease. Patients were aged between 15 and 75 years with Eastern Cooperative Oncology Group Performance Status (ECOG PS) of ≤ 2; with normal pre-treatment haematological and biochemical function and radiologically measurable disease. Patients who were pregnant or lactating, harbouring active infection, had a history of hypersensitivity to taxanes or exposed previously to gemcitabine and/or docetaxel, were excluded.

The histological diagnosis was confirmed by two expert sarcoma pathologists at our institution (A.B. and A.M.). The study was carried out in accordance with Good Clinical Practice Guidelines after approval by the Institutional Review Board and provision of informed consent by the patient or their legal guardian (in patients aged less than 18 years).

### Study procedures and schema

The baseline investigations consisted of complete hemogram, organ function tests, Lactate dehydrogenase (LDH) and radiological imaging (Contrast Enhanced CT or FDG PET scan). Formalin-fixed paraffin embedded (FFPE) tissue was utilized for performing translocation (x;18) test by Break-Apart Fluorescent In-Situ Hybridization (FISH) assay for the SS18 and its partner genes (SSX1 and SSX2). Translocation positivity was not essential for enrolment if the diagnosis was confirmed by the sarcoma pathologists.

The treatment consisted of 12 weeks of GD combination with gemcitabine 900 mg/m^2^ (10 mg/m^2^/minute) on days 1 and 8 and docetaxel 75 mg/m^2^ on day 8 in a 21-day cycle with Granulocyte Colony Stimulating Factor (G-CSF) support for 5 days from day 9. Dose modification at baseline was performed for amputees [13] and patients with exposure to radiotherapy to the flat bones (25% dose reduction). Toxicity analysis was done prior to days 1 and 8 of the chemotherapy cycles as per the Common Toxicity Criteria for Adverse Events (NCI CTCAE v5.0) and dose modifications (25% reduction per level) were made in accordance with the study protocol described in Additional file 1. Patients who had unacceptable toxicity as per protocol, failure to comply with the study regimen and withdrawal of consent would be removed from the study. Tumor response was assessed clinically at each hospital visit prior to chemotherapy, and as per the Response Evaluation Criteria in Solid Tumors (RECIST) version 1.1 at week 12 [14]. Patients who did not progress at the end of 12 weeks were continued on the same regimen outside the study up to a maximum of 6 cycles. All patients were followed up for survival outcomes, until death or withdrawal of consent.

Quality of life (QoL) assessment was done by the 30-item questionnaire developed by European Organization for Research and Treatment of Cancer (EORTC)—the Core Quality of life Questionnaire (QLQ C30) [15]. An absolute difference of more than 10 points between the QoL scores at 12 weeks and baseline was clinically significant [16]. A higher functional scale and global health status (GHS) score represents a better QoL, while a higher symptom scale score represents higher symptom burden and worse QoL.

### Study outcomes

The primary end point was 3-month Progression-free Rate (PFR) and the secondary end points included OS, Progression Free Survival (PFS), Response Rates (RR), toxicity profile and QoL analysis. 3-month PFR was defined as the proportion of patients who were free of disease progression at the end of 12 weeks. OS was defined as the time from randomization to occurrence of death due to any cause/or to the date of censoring at the last time the subject was known to be alive. PFS was the time between treatment initiation and tumor progression or death from any cause. The patient reported outcome (PRO) in terms of QoL was assessed at baseline and week 12.

### Statistical methods and sample size calculation

Van Glabbeke et al. suggested the 3-month PFR for the second-line treatment to represent an active agent to be more than 39%, while that for an inactive one would be less than 21% [17]. For the sample size calculation, Simon optimal one-sample, two-stage testing was applied. The null hypothesis tested the true value of 3-month PFR was 20% with gemcitabine docetaxel combination in relapsed metastatic/locally advanced unresectable synovial sarcoma against the alternative hypothesis of the 3-month PFR being 40%. Type 1 (alpha) error of 0.1 and type 2 (beta) error of 0.2, 40% PFR of gemcitabine-docetaxel combination (p1) and 20% PFR of standard therapy (p0) were used to compute the sample size, which was estimated to be 43. In the first stage, 13 patients were to be recruited and the study could proceed to the second stage if at least 3 patients were free from progression. At the end of stage 2, at least 12 responses would be required for the study to warrant further investigation.

### Statistical analysis

The statistical computations of the intention-to-treat (ITT) population were performed by SPSS version 26.0. Descriptive statistics (mean, median and range) were calculated for all variables. The Kaplan–Meier method was used for estimation of survival measures (median OS and PFS) along with 95% confidence interval (CI). Differences between survival measures among subgroups were estimated using the univariate Cox model and significant factors would be included in a multivariate Cox model, with P values of < 0.05 considered statistically significant. 3-month PFR, response rates by i.e. Complete Response (CR), Partial Response (PR), Stable Disease (SD), Overall Response Rates (ORR) were calculated with 95% CI based on exact binomial distribution.

## Results

### Patient characteristics

**Fig. 1:**
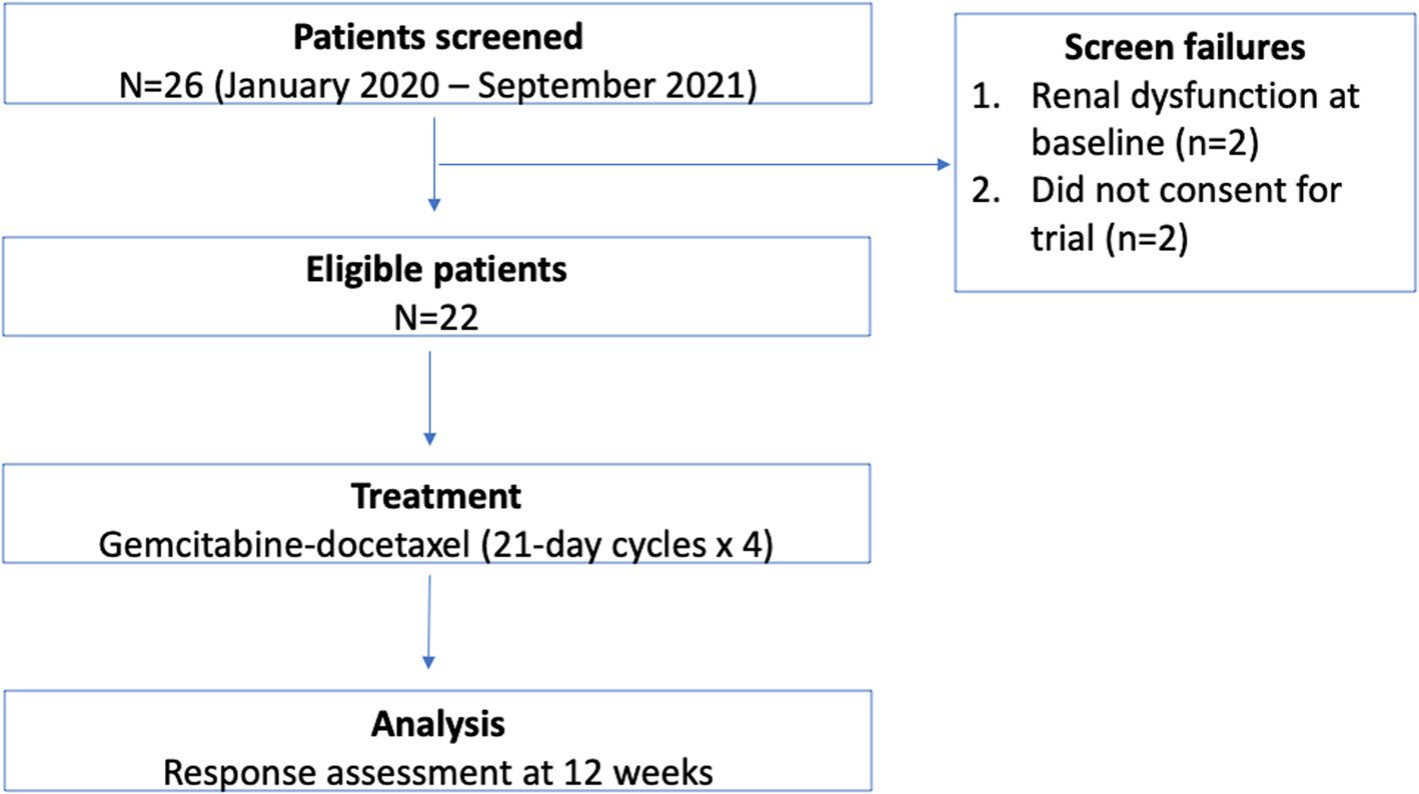
Consolidated standards of reporting trials diagram portraying the trial profile

Twenty-two patients were enrolled in this study out of the 26 patients screened, following which the study was closed early due to slow accrual (Fig. 1). Out of the 22 patients, 13 (59%) were male and the median age was 32 years (range, 15–60 years). The baseline ECOG PS was 0–1 in 13 (59%) patients and 2 in the remaining 9 (41%). Break-apart FISH SYT-SSX testing was performed in 20 (90.8%) patients, out of whom 17 (77.2%) were positive. 18 (81.8%) patients had metastatic disease while the remaining 4 (18.2%) had locally advanced, unresectable disease at enrolment. The most common primary sites of disease were extremity in 15 (68%), trunk in 3 (13.6%) and visceral organs in 2 (9%). 17 (77.2%) patients had undergone surgery during their treatment course while 11 (50%) and 2 (9%) patients had received radiotherapy in the curative and palliative setting respectively. The median number of lines of treatment received prior to enrolment was 1 (range 1–4) with all exposed to anthracyclines, and 8 (36.4%) patients exposed pazopanib. 10 (45.4%) patients had received previous treatment in the form of (neo)adjuvant therapy and the remainder as palliative therapy. Further details of the baseline characteristics and previously received treatments by the study population are summarized in Tables 1 and 2.

**Table 1:** Clinical profile and disease characteristics of patients in the study

Parameter	Values
**Median age, years (range)**	32 (15–60)
**Sex, n (%)**	
Males	13 (59)
Females	9 (41)
**ECOG PS, n (%)**	
1	13 (59)
2	9 (41)
**Primary location of disease, n (%)**	
Extremity	16 (72.7)
Trunk	3 (13.6)
Viscera	1 (4.5)
Head and neck	1 (4.5)
Paraspinal	1 (4.5)
**Stage at enrolment**	
Metastatic	18 (81.8)
Locally advanced unresectable	4 (18.2)
**Metastatic sites, n (%)**	
Pulmonary	18 (81.8)
Pleura	3 (13.6)
Subcutaneous	3 (13.6)
Lymph nodal	2 (9)
Skeletal	1 (4.5)
Adrenal	1 (4.5)
**Translocation (x; 18), n (%)**	
Positive	17 (77.2)
Negative	3 (13.6)
Not performed	2 (9)
**Median albumin, g/l (range)**	4.2 (3.2–5.2)
**Median LDH (range)**	232 (186–1186)
**Median NLR (range)**	2.2 (1.07–8.50)

*PS* Performance Status, *LDH* Lactate Dehydrogenase, *NLR* Neutrophil Lymphocyte Ratio

**Table 2:** Treatment modalities received by patients prior to enrolment in the study

Parameter	N (%)
**Median lines of previous therapy, n (range)**	1 (1–4)
**Best response to previous line of chemotherapy, n (%)**	
Partial response	7 (31.8)
Stable disease	7 (31.8)
Progressive disease	8 (36.4)
**Duration of response after first line of therapy**	
Less than 6 months	9 (41)
Greater than 6 months	13 (59)
**Types of medical therapy received prior to enrolment, n (%)**	
First line	*N* = 22
Anthracycline-based	22 (100)
Second Line	*N* = 11
Pazopanib	7 (63.6)
High-dose ifosfamide	3 (27.2)
Ifosfamide-Cisplatin-Paclitaxel	1 (4.5)
Third line	*N* = 3
Pazopanib	1 (33.3)
High-dose ifosfamide	1 (33.3)
Tazemetostat	1 (33.3)
Fourth line	*N* = 2
Regorafenib	1 (50)
Trabectedin	1 (50)
**Previous surgical therapy, n (%)**	
Amputation	3 (13.6)
Limb salvage	14 (63.6)
None	5 (27.2)
**Previous radiation therapy, n (%)**	
Post-operative	9 (41)
Neoadjuvant	2 (9)
Palliative	2 (9)

### Study treatment and efficacy

**Fig. 2:**
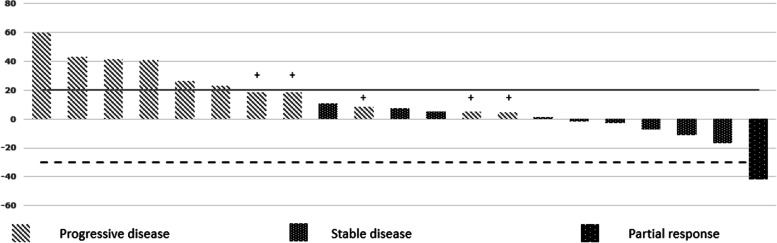
Waterfall plot representing the percentage maximum tumor reduction at 12 weeks of treatment according to RECIST version 1.1. [Patients who had disease progression based on development of new lesions without increase in dimensions are labelled with “ + ”]

**Fig. 3:**
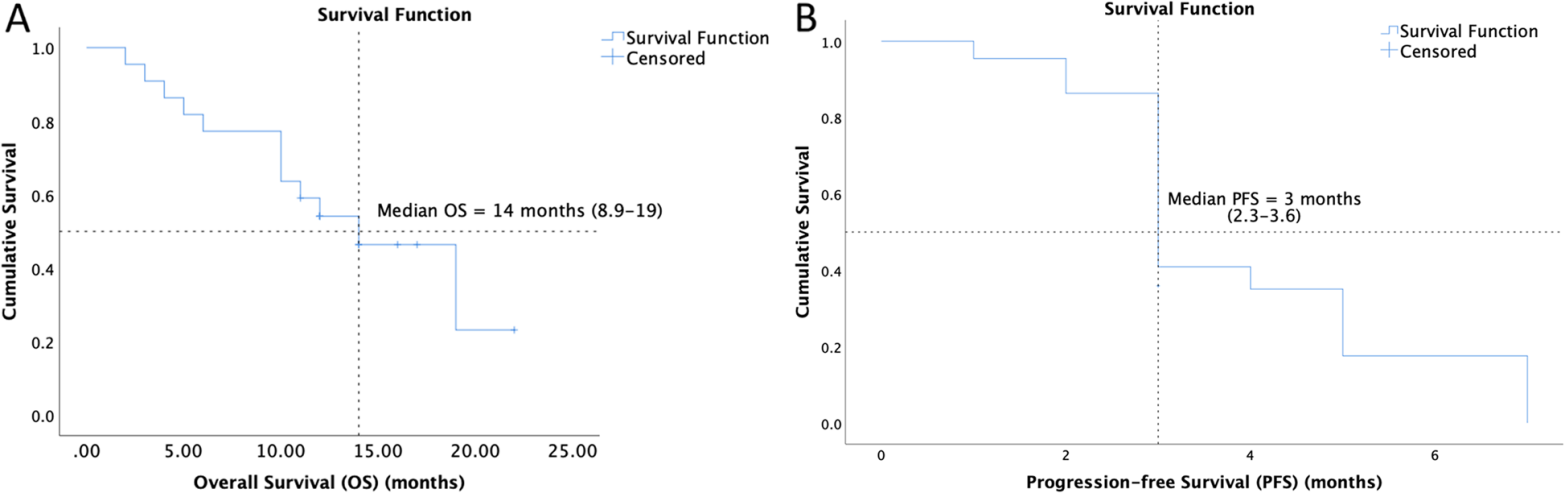
Kaplan–Meier analyses of (**A**) overall survival and (**B**) progression-free survival of patients treated with gemcitabine docetaxel combination. OS: Overall survival; PFS: progression-free survival

Gemcitabine at median dose of 1400 mg (range 1000–1700 mg) and docetaxel at median dose of 120 mg (range 90–140 mg) were administered for a median of 4 cycles (range 2–4) during the study period of 12 weeks. Overall, the median number of cycles received for the entire course of treatment was 4 cycles (range 2–6). Reduction of the starting dose was required in 4 (18.1%) patients due to amputation in 3 (13.6%) and prior exposure to spinal radiotherapy in 1 (4.5%) patient. 3 (13.6%) patients required dose reductions during the therapy, at median 25% (range 25–50%) with the most common indication being grade 3 oral mucositis (2, 9%). The median starting intensity of chemotherapy was 100% (range 75–100) and the lowest dose received was 50% in 1 patient. 7 (31.8%) patients experienced delays in their chemotherapy schedule for a median of 7 days (range 4–17 days), most frequently due to hematological toxicity. The 3-month PFR was 45.4% (95% CI 24.8–66.1%) with 10 patients free of progression at the end of the study period. Of these, 9 patients (41%) attained SD and 1 patient (4.5%) had PR to the treatment at 12 weeks (Fig. 2). The 6-month PFR was 9.09%. At a median follow-up of 14 months (95% CI 10.8–17.1), the median PFS was 3 months (95% CI 2.3–3.6) and median OS was 14 months (95% CI 8.9–19) (Fig. 3). Subgroup analyses on the basis of gender, ECOG PS, albumin, Neutrophil–Lymphocyte Ratio (NLR), relapse-free interval post 1^st^ line of therapy and number of previous lines of therapy, did not yield any significant association with survival outcomes. The outcomes of subgroup analysis for OS and PFS have been detailed in Additional file 2.

Multiple patients received more than 1 line of therapy after completion of the study period including 7 (31.8%) who completed total 6 cycles of GD. The other treatments administered after the study were pazopanib (10, 45.4%), regorafenib (5, 22.7%), ifosfamide (3, 13.6%), temozolomide (2, 9%) and anlotinib (2, 9%). 1 patient (4.5%) underwent enrolment in a phase I/II clinical trial of epigenetic modifier-based therapy.

### Adverse event profile

Twenty-one patients (95.4%) experienced adverse effects during the study regimen, including 7 (31.8%) with grade 3 or worse adverse events (Table 3). The most common haematological all-grade toxicities included anemia in 17 (77%), thrombocytopenia in 4 (18%) and neutropenia in 3 (13.6%) patients. The most frequent non-hematological all-grade toxicities included fatigue in 17(77%), transaminitis in 16 (72.7%) and vomiting in 7 (31.8%) patients. The most common grade 3 or worse toxicities included anemia in 4 (18%), neutropenia in 2 (9%) and thrombocytopenia in 2 (9%) patients. There were 7 mortalities among the ITT population which included deaths due to disease progression in 6 (27.2%) patients and accidental death in 1 (14.2%) patient. Among the former, 1 patient developed decompensated liver failure due to acute Hepatitis B viral infection and died due to a combination of disease progression and viral hepatitis.

**Table 3:** Profile of the adverse events noted in the study population

Adverse event, n (%)	All grades	Grade ≥ 3
Any adverse event	21 (95.5)	7 (31.8)
**Hematological**		
Anemia	17 (77.2)	4 (18.1)
Neutropenia	3 (13.6)	2 (9)
Thrombocytopenia	4 (18.1)	2 (9)
**Non-hematological**		
Nausea/vomiting	7 (31.8)	0 (0)
Fatigue	17 (77)	0 (0)
Mucositis/stomatitis	5 (22.7)	1 (4.5)
Diarrhea	5 (22.7)	0 (0)
Cutaneous toxicity	4 (18.1)	0 (0)
Infections (Non-neutropenic)	3 (13.6)	1 (4.5)
Alopecia	5 (22.7)	0 (0)
Thrombophlebitis	3 (13.6)	0 (0)
Jaundice	1 (4.5)	1 (4.5)
Elevated transaminases	16 (72.7)	1 (4.5)

### QoL analysis

**Fig. 4:**
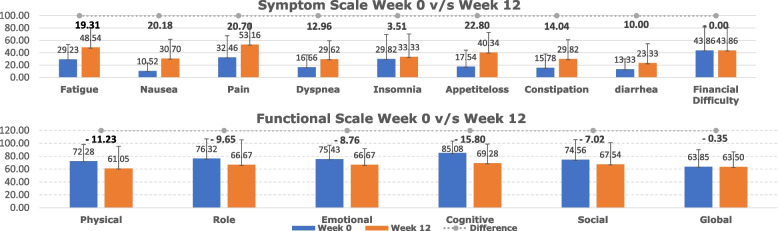
Bar graph showing differences in various parameters of QLQ C-30 domains at 0 and 12 weeks with statistically significant worsening denoted by “ + ”

The EORTC QLQ C30 questionnaire was filled by 100% patients at baseline and 18 (81.8%) at week 12. The absolute mean difference between QoL measures at 0 and 12 weeks showed clinically significant worsening (that is, a difference of more than 10 points) among functional (physical, emotional, role and cognitive functioning) and symptom (fatigue, nausea/vomiting, pain, dyspnea, loss of appetite, constipation, diarrhea) scales. This translated into statistically significant worsening among the emotional, loss of appetite, nausea/vomiting and fatigue parameters which have been detailed in Additional file 3. The maximum decline was in “loss of appetite”, with a mean score difference of 25.9 (95% CI 8.3–43.5) between 0 and 12 weeks. The financial and GHS remained both numerically and statistically stable between baseline and 12 weeks (Fig. 4).

## Discussion

In this first prospective trial on GD in patients with metastatic/unresectable locally advanced relapsed SS, we show the meaningful activity of this combination.

The benefit of GD in advanced STS has been previously demonstrated by Maki et al. with a median PFS and OS benefit of 4.2 months and 6.4 months respectively compared to gemcitabine alone [18]. This superior survival outcome was shown particularly among leiomyosarcoma and Undifferentiated Pleomorphic Sarcoma, and the representation of SS in this study was less than 10%. Patients with SS constituted 4% of the GD arm in the phase 3 GeDDiS trial, making the interpretation of its activity in SS difficult [19]. The only study to address the efficacy of GD in SS alone was a retrospective analysis of 22 patients, with ORR of 5% and median PFS of 2 months [20].

The 12-week PFR of 45.4% meets the primary endpoint in our study, and supports the utility of GD as a treatment option in patients with advanced, previously treated SS. While the 12-week PFR and median PFS were 63.8% of 5.9 months respectively in the frontline GeDDiS trial, our study yielded a median PFS of 3 months. However, our patients received the regimen in the pre-treated setting, even including 11 (50%) who had already received 2 lines of treatment.

We found that the GD combination had a manageable toxicity profile in this cohort. Though grade 3 (or worse) toxicities were documented in a total of 7 (31.8%) patients, there were no episodes of febrile neutropenia or bleeding. The acute liver failure with hyperbilirubinemia and transaminitis noted in 1 patient occurred due to acute Hepatitis B infection, which led to mortality. 3 (13.6%) patients underwent chemotherapy dose reductions in our study, which contrasts with the high frequency of dose reductions (46%) and discontinuations (40%) reported by Maki et al., with the doses of gemcitabine (900 mg/m^2^) and docetaxel (100 mg/m^2^). These findings demonstrate the tolerability of gemcitabine 900 mg/m^2^at FDR in most patients of our study. Serious adverse events associated with another regimen utilised in SS, high-dose ifosfamide, include infection (16%), febrile neutropenia (39%), acute renal failure (2%) and neurotoxicity (11%) [21]. GD is comparatively associated with a more manageable toxicity profile than agents such as high-dose ifosfamide.

The deterioration in multiple functional and symptom scales of the QoL assay underlines the need for better supportive care interventions that could help mitigate some contributing factors. The administration of this regimen has been considered relatively difficult due to the more frequent and longer hospital visits required [19]. However, the stable global health and financial scale parameters in our study are encouraging outcomes, especially in this population of pre-treated patients. The GeDDiS trial had also compared QoL measures at 12 weeks between GD and doxorubicin, and had not found any statistically significant difference [19]. The QoL outcomes at 4, 8 and 12 weeks reported by the PALETTE trial did not reach the significant 10-point difference between pazopanib and placebo in terms of the GHS. Symptom scale parameters such as diarrhea, loss of appetite, nausea/vomiting and fatigue were significantly worse in the pazopanib arm compared to placebo [22].

Anthracyclines continue to be the standard first-line therapy for advanced SS based on previous literature [23] and GD has not been found superior to frontline doxorubicin in patients with SS [19]. The non-doxorubicin anthracycline formulations that have been used as first-line therapies in patients with advanced STS include non-pegylated liposomal doxorubicin (PLD) and epirubicin [24]. A phase II trial among 34 patients with metastatic STS (including 4 with synovial sarcoma) reported ORR of 55.9%, median PFS of 4.2 months and 3% symptomatic grade 3 cardiotoxicity with the combination of ifosfamide and PLD [25]. Among the therapies used for SS in the relapsed setting, high-dose ifosfamide has produced ORR of 44% and median PFS of 11.6 months in a retrospective study in metastatic pre-treated SS [26]. Trabectedin has shown activity in a pooled analysis of phase 2 trials on translocation-related sarcomas, depicting a median PFS of 4.6 months and ORR of 4% [27] while an Italian multicentre analysis found tumor control rate of 50% in SS [28]. Pazopanib exhibited 12-week PFR of 49% among the SS cohort in a phase 2 trial [29], and a median PFS of 4.6 months in a phase 3 study with significant survival benefit in the SS subgroup [30]. Among other multikinase inhibitors, regorafenib prolonged the median PFS in a placebo-controlled trial (5.6 versus 1.0 months) while sorafenib has shown limited results [31, 32]. Anlotinib, a novel multikinase inhibitor, has shown promising results in SS, with a median PFS of 2.89 months, 6-month PFR 42.3% and 1-year PFR of 26.9% in a phase 3 study [33]. Table 4 describes the results obtained from our study in comparison with outcomes with other active agents used in patients with relapsed, advanced SS.

**Table 4:** Summary of outcomes and toxicity profile of other treatment options for relapsed synovial sarcoma in comparison to the study regimen

Treatment	Median Progression-free survival (PFS)	Median Overall survival (OS)	Common adverse events associated with drug (CTCAE Grade ≥ **3)**
Study regimen (Gemcitabine Docetaxel)	3 months (95% CI: 2.3–3.6)	14 months (95% CI 8.9–19)	Anemia (18%) Neutropenia (9%) Thrombocytopenia (9%)
High-dose ifosfamide [26]	11.6 months (95% CI: 9–14)	NA	Neutropenia (47%) Febrile neutropenia (12%) Thrombocytopenia (12%) Cystitis (3%) Neurological side effects (3%)
Trabectedin [34, 35]	3.8 months (95% CI: 3.2–4.7)	10.4 months (95% CI: 8.5–12.7)	Neutropenia (43%) Thrombocytopenia (21%) Anemia (19%)
Pazopanib [30, 35],	5.3 months (95% CI: 4.2–6.7)	10.3 months (95% CI 8.4–12.6)	Fatigue (13%) Hypertension (7%) Diarrhea (5%)
Regorafenib [31]	5.6 months (95% CI1.4–11.6)	13.4 months (95% CI: 5.3-NR)	Hypertension (18%) Hand foot skin reaction (15%) Asthenia (13%)
Anlotinib [33]	2.89 months (95% CI: 2.73–6.87)	NA	Diarrhea (5.8%) Hypertension (3.8%)
Tazemetostat [36]	4-month PFR: 15%	NA	Cough^a^ (36%) Dyspnea^a^ (33%) Fatigue^a^ (33%)

*PFR* Progression-free survival rate, *CI* Confidence interval, *NR* Not reached, *NA* Not available, *CTCAE* Common Toxicity Criteria for Adverse Events

^a^Only grade 1/2 events reported in the study among patients with synovial sarcoma

CTAs such as NY-ESO-1, PRAME, MAGEA4 and MAGEA1 represent newer therapeutic targets due to their high expression in SS [8]. The prospects for CTA-based therapy appear exciting with targeted vaccines and autologous T-cell receptor (TCR) therapies. While NY-ESO-1^c259^- based T-cell therapy yielded median duration of response of 7.7 months and anti-tumor responses in 50%, MAGE-A4 produced a durable response rate of 44% among 7 patients with SS [37, 38]. The challenge with TCR therapies is the long processing time after leukapheresis and utility only in patients expressing HLA A*02:01. This expression is lower among Asians and African-Americans in comparison to Caucasians [39] creating an unmet need for later-line therapies.

The addition of other active agents with non-overlapping toxicity profiles to the GD backbone warrants exploration. Olaratumab added to GD regimen in the second-line treatment of advanced STS produced no significant OS benefit but a clinically meaningful benefit in PFS and ORR in a phase 1b/2 study [40]. Future trials incorporating targeted agents such as tazemetostat to GD might be conducted among patients with relapsed advanced SS.

Even though GD has lesser response rates and survival outcomes compared to certain therapies such as anlotinib, it can be considered as a treatment option for those patients with relapsed advanced SS. Patients who especially stand to benefit from our findings include those who do not have to access to newer therapies such as anlotinib and cancer vaccines or are ineligible for adoptive immunotherapy due to absence of the HLA-A*02:01 allele. The limitations of our study include the constraints of a single-arm design and the planned sample size not being met owing to slowed accrual during the COVID-19 pandemic. It is still noteworthy that this is the only prospective study of GD to focus on SS and to demonstrate the efficacy and acceptable toxicity profile of this combination.

## Conclusion

This study suggests the potential use of GD as a treatment in relapsed metastatic/locally advanced unresectable SS, especially in populations lacking access to, or ineligible for novel therapeutic options. Collaborative efforts are warranted in the future to assess the efficacy of this regimen in a larger cohort of patients.

## Supplementary Information

**Additional file 1: Table A.1.** Modification of trial regimen according to chemotherapy-related toxicity.

**Additional file 2: Table A.2. **Description of results of subgroup analysis of Overall Survival and Progression-free Survival.

**Additional file 3: Table A.3. **Difference in mean quality of life (QoL) scores between 12 weeks and baseline.

## Data Availability

The datasets used and/or analysed during the current study available from the corresponding author on reasonable request.

## Abbreviations

SS: Synovial sarcoma
OS: Overall survival
TNT: Time to next treatment
CTA: Cancer testis antigen
GD: Gemcitabine docetaxel
FDR: Fixed dose rate
ECOG PS: Eastern Cooperative Oncology Group Performance Status
LDH: Lactate dehydrogenase
FFPE: Formalin-fixed paraffin embedded
FISH: Fluorescent In-Situ Hybridization
G-CSF: Granulocyte Colony Stimulating Factor
CTCAE: Common Toxicity Criteria for Adverse Events
RECIST: Response Evaluation Criteria in Solid Tumors
QoL: Quality of Life
EORTC: European Organization for Research and Treatment of Cancer
QLQ-C30: Core Quality of life Questionnaire
GHS: Global health status
PFR: Progression-free rate
PFS: Progression-free Survival
RR: Response rate
PRO: Patient reported outcomes
CI: Confidence interval
PFR: Progression-free Rate
ITT: Intention to treat
TCR: T-cell receptor
