# Supplementary material for: A phase II study of gemcitabine and docetaxel combination in relapsed metastatic or unresectable locally advanced synovial sarcoma
Source: BMC Cancer. 2023 Jul 8;23:639. doi: 10.1186/s12885-023-11099-4 (PMC10329387; doi:10.1186/s12885-023-11099-4)
Supplement: Supplementary file 1 — Additional file 1: Table A.1. Modification of trial regimen according to chemotherapy-related toxicity. [file 12885_2023_11099_MOESM1_ESM.docx]

| PARAMETER | TIMING | TOXICITY | CTCAE GRADE (1-5) | ACTION WITH STUDY TREATMENT |
| --- | --- | --- | --- | --- |
| Hematological | | | | |
| Neutrophils  (x10^9^/L) | Previous cycle | ANC less than 0.5/L for ≥7days | 4 | Delay D1 until resolved and  reduce doses by 1 level (25%). |
|  |  | febrile neutropenia, or infection with neutropenia | 4 |  |
|  | Day 1 | ANC 1.0-1.5/L | 2 | Delay D1 until >1.5 AND  If delay <15d, reduce doses by 1 level.  If delay ≥15d, discontinue. |
|  |  | ANC <1.0/L | 3-4 | Delay D1 until >1.5 AND  reduce doses by 1 level. |
|  | Day 8 | ANC <1.0/L | 3-4 | Omit D8 AND  reduce doses by 1 level. |
| Platelets  (x10^9^/L) | Previous cycle | <25/L or bleeding | 4 | Delay D1 until resolved AND  reduce doses by 1 level |
|  | D1 | <100/L | 1-4 | Delay D1 until >99 AND  If delay <15d, reduce doses by 1 level  If delay ≥15d, discontinue. |
|  | D8 | <75/L | 2-4 | Omit D8 AND  reduce doses by 1 level. |
| Haemoglobin (g/L) | First occasion | <90 | 2 | Transfuse to Hb >100 AND treat as scheduled. |
|  | Second occasion | <90 | 2 | Transfuse to Hb >100, treat as scheduled, AND reduce doses by 1 level. |
| Non-hematologic toxicity* | | | | |
| Hepatic System | Bilirubin | >3.0 ULN | 3-4 | Discontinue |
|  | AST/ALT | >5.0 x ULN AND  ≥ 2 x baseline | 3-4 |  |
| Renal | Creatinine | Severe toxicity or HUS | 2-4 | Withhold and delay till Grade 1  As indicated, dialysis: 6-12hrs after chemotherapy |
| Bilirubin | Jaundice | >3.0 x ULN | 3-4 | Discontinue |
| Infection |  |  | 2-4 | Delay until Grade 0-1 Restart when Grade 0-1  Reduce doses by 1 level |
| GI | Nausea |  | 3-4 | Delay until Grade 0-1 |
|  | Vomiting |  | 3-4 | Restart when Grade 0-1 |
|  | Diarrhoea |  | 3-4 | Restart when Grade 0-1  Reduce doses 1 level |
|  | Oral mucositis |  | 3-4 | Restart when Grade 0-1  Reduce doses 1 level |
| Cardio-vascular | Myocardial infarction |  |  | Discontinue |
| Vascular | Thombo-embolic event | Venous (DVT, PTE) | 3-4 | Delay until adequately treated Restart at physician discretion  Anticoagulate with heparin, not warfarin, whilst on study drug |
|  |  | Arterial | 3-4 | Discontinue |
| Skin | Various |  | 3-4 | Delay until Grade 0-1 Restart when Grade 0-1  Reduce subsequent doses 1 level |
| *Delay chemotherapy till resolution of toxicity till grade 0-1  Day 1 treatment may be delayed for a maximum of 14 days. If the adverse event has not resolved to G0-1 after delaying day 1 treatment for 14 days, then chemotherapy should be discontinued. | | | | |

Table A.1: Modification of trial regimen according to chemotherapy-related toxicity.

Abbreviations - ANC: Absolute neutrophil count, CTCAE: Common Terminology Criteria for Adverse Events, Hb: Hemoglobin, AST: Aspartate transaminase, ALT: Alanine transaminase, DVT: Deep vein thrombosis, PTE: Pulmonary thromboembolism, ULN: Upper limit of normal
