# Supplementary material for: A phase II study of gemcitabine and docetaxel combination in relapsed metastatic or unresectable locally advanced synovial sarcoma
Source: BMC Cancer. 2023 Jul 8;23:639. doi: 10.1186/s12885-023-11099-4 (PMC10329387; doi:10.1186/s12885-023-11099-4)
Supplement: Supplementary file 2 — Additional file 2: Table A.2. Description of results of subgroup analysis of Overall Survival and Progression-free Survival. [file 12885_2023_11099_MOESM2_ESM.docx]

|  | PFS | | OS | |
| --- | --- | --- | --- | --- |
| Variable | Univariate analysis | | Univariate analysis | |
|  | HR (95%CI) | p value | HR (95%CI) | p value |
| Gender   - male - female | 0.185 (0.025-1.344) | 0.09 | 1.48 (0.553-3.972) | 0.43 |
| Primary site   - extremity - non-extremity | 1.08 (0.248-4.734) | 0.91 | 1.12 (0.192-6.55) | 0.89 |
| Stage at baseline   - locally advanced - metastatic | 0.083 (0.002-3.414) | 0.19 | 2.82 (0.2-38.1) | 0.43 |
| ECOG PS   - 0-1 - 2 | 3.58 (0.31-40.2) | 0.30 | 0.921 (0.165-5.15) | 0.92 |
| Serum Albumin   - ≤3.5 mg/dl - >3.5 mg/dl | 0.08 (0.002-3.13) | 0.17 | 0.259 (0.02-2.95) | 0.27 |
| NLR   - ≤1.7 - >1.7 | 0.728 (0.13-4.03) | 0.71 | 2.1 (0.28-15.7) | 0.45 |
| Relapse-free interval post 1^st^ line of treatment   - ≤6 months - >6 months | 0.96 (0.36-2.61) | 0.94 | 1.13 (0.16-7.75) | 0.89 |
| Lines of treatment   - ≤2 - >2 | 1.67 (0.39-7.08) | 0.48 | 2.27 (0.48-10.63) | 0.29 |

Table A.2: Description of results of subgroup analysis of Overall Survival and Progression-free Survival

Abbreviations - OS: Overall Survival, PFS: Progression-free survival
