# Supplementary material for: A phase II study of gemcitabine and docetaxel combination in relapsed metastatic or unresectable locally advanced synovial sarcoma
Source: BMC Cancer. 2023 Jul 8;23:639. doi: 10.1186/s12885-023-11099-4 (PMC10329387; doi:10.1186/s12885-023-11099-4)
Supplement: Supplementary file 3 — Additional file 3: Table A.3. Difference in mean quality of life (QoL) scores between 12 weeks and baseline. [file 12885_2023_11099_MOESM3_ESM.docx]

| C30 score parameters | Mean difference between week 0 and week 12 | SD | SE Mean | Lower | Upper | P value |
| --- | --- | --- | --- | --- | --- | --- |
| Global Health Status* | - 0.3 | 29.3 | 6.7 | -13.7 | 14.4 | 0.052 |
| Symptom scales** | | | | | | |
| Fatigue | -19.2 | 30.0 | 6.8 | -33.7 | -4.8 | 0.012 |
| Dyspnea | -12.9 | 25.9 | 6.1 | -25.8 | -0.07 | 0.049 |
| Appetite loss | -22.8 | 36.9 | 8.4 | -40.6 | -5.0 | 0.015 |
| Nausea/vomiting | -20.1 | 29.6 | 6.8 | -34.4 | -5.8 | 0.008 |
| Pain | -20.7 | 33.5 | 7.6 | -24.9 | 7.3 | 0.269 |
| Insomnia | -3.5 | 47.0 | 10.7 | -26.1 | 19.1 | 0.749 |
| Appetite loss | -22.8 | 36.9 | 8.4 | -40.6 | -5.0 | 0.15 |
| Constipation | -14.0 | 37.3 | 8.5 | -32.0 | 3.9 | 0.119 |
| Diarrhea | -10.0 | 32.6 | 7.2 | -25.2 | 5.2 | 0.186 |
| Financial difficulties | 0 | 40.0 | 9.1 | -19.3 | 19.3 | 1.00 |
| Functional scales* | | | | | | |
| Physical functioning | 11.2 | 29.2 | 6.7 | -2.8 | 25.3 | 0.111 |
| Role functioning | 9.6 | 27.9 | 6.4 | -3.8 | 23.1 | 0.150 |
| Emotional functioning | 8.7 | 21.6 | 4.9 | -1.6 | 19.1 | 0.094 |
| Cognitive functioning | 15.8 | 33.5 | 7.7 | -.37 | 31.9 | 0.055 |
| Social functioning | 7.0 | 29.0 | 6.7 | -13.7 | 14.4 | 0.959 |

Table A.3: Difference in mean quality of life (QoL) scores between 12 weeks and baseline.

*Higher score is associated with better QoL; positive difference of scores between baseline and 12 weeks is representative of a better functioning and QoL

**Higher score is associated with worse QoL; negative difference of scores between baseline and 12 weeks is representative of a worsening symptom burden and QoL

SD: standard deviation; SE: standard error.
